# The Role of Immature Granulocyte Percentage and Other Inflammatory Hematological Markers in Predicting In Vitro Fertilization Success

**DOI:** 10.3390/biomedicines13112819

**Published:** 2025-11-19

**Authors:** Dilay Gök Korucu, Emre Uysal, Fatih Akkuş, Pınar Tombaklar, Şükran Doğru, Oğuzhan Günenç

**Affiliations:** 1 IVF Unit, Department of Obstetrics and Gynecology, Konya City Hospital, Health Sciences University, Konya 42020, Turkey; 2 Department of Obstetrics and Gynecology, Yusufeli State Hospital, Artvin 08800, Turkey; emreuysal53@dr.com; 3 Division of Perinatology, Department of Obstetrics and Gynecology, Meram Medical Faculty, Necmettin Erbakan University, Konya 42090, Turkey; fakkus1987@gmail.com; 4 Department of Clinical Biochemistry, Konya City Hospital, Health Sciences University, Konya 42020, Turkey; pinartonbaklarbilgi@gmail.com; 5 Division of Perinatology, Department of Obstetrics and Gynecology, Konya City Hospital, Health Sciences University, Konya 42020, Turkey; sukrandogru-2465@hotmail.com; 6 Department of Obstetrics and Gynecology, Konya City Hospital, Health Sciences University, Konya 42020, Turkey; oguzhangunenc@hotmail.com

**Keywords:** complete blood count, immature granulocyte percentage, inflammatory markers, IVF success, pregnancy outcome

## Abstract

**Background:** This study explores the role of inflammatory hematological markers from complete blood count (CBC) parameters in predicting the in vitro fertilization (IVF) treatment success of patients. It focuses particularly on the immature granulocyte (IG) percentage, a novel inflammatory marker. To our knowledge, this is the first study in the literature examining the relationship between IG percentage and pregnancy outcomes in IVF. **Methods:** Conducted retrospectively, this study included 311 IVF cycles from 311 distinct patients. A binomial logistic regression model identified factors affecting pregnancy outcomes. Various predictors were analyzed, including embryo quality, white blood cell (WBC) count, IG percentage, systemic inflammatory index (SII), endometrial thickness on the day of human chorionic gonadotropin (hCG) administration, number of mature (MII) oocytes, and the number of embryos generated. **Results:** The IG percentage significantly impacted clinical pregnancy and live birth outcomes (OR = 0.40, 95% CI (confidence interval): 0.317–0.51, *p* < 0.001), unlike other inflammatory markers. Each millimeter increase in endometrial thickness measured on the day of hCG administration was associated with a slight increase in the likelihood of IVF success (OR: 1.20, 95% CI 1.004–1.44, *p* = 0.044). Neither SII nor mature oocyte count significantly affected pregnancy outcomes. **Conclusions:** A low IG percentage correlates positively with clinical pregnancy and live birth outcomes. Measuring IG percentage offers a rapid, inexpensive, and readily available method to predict IVF success.

## 1. Introduction

The question of predictive markers of IVF success has led researchers to explore various physiological and immunological factors. Among these, hematological inflammatory markers have emerged as potential indicators of IVF outcomes. However, while some studies [1,2] have delved into common markers such as the neutrophil-to-lymphocyte ratio (NLR) and platelet-to-lymphocyte ratio (PLR), the role of IG percentage in this context remains largely unexplored. IGs are young forms of white blood cells that are part of the body’s immune system, specifically within the granulocyte class, including neutrophils, eosinophils, and basophils. These cells are typically found in the bone marrow and are released into the bloodstream in response to infection or inflammation [3].

Elevated IG percentages reflect the premature release of granulocytes from the bone marrow in response to systemic inflammatory stimuli, which may also mirror the immune–inflammatory state of the endometrium. This immunological imbalance could interfere with implantation and early embryo development.

Inflammatory processes influence reproductive biology significantly, impacting implantation, embryo development, and pregnancy maintenance [4]. Markers like NLR and PLR, commonly derived from CBC parameters, have been associated with systemic inflammation and studied in various medical conditions, including obstetric and gynecological disorders. For instance, Sisti et al. investigated the relationship between such markers and HELLP syndrome, an inflammatory obstetric condition, providing insights into the complex interplay between inflammation and pregnancy outcomes [5]. In another study, higher levels of IGs were observed in pregnant women with hepatic cholestasis compared to normal pregnant women and were considered an indicator of inflammation [6].

Recently, researchers have begun to examine these inflammatory hematological parameters more closely in the context of IVF outcomes. For example, Li et al. reported that elevated systemic immune–inflammation (SII) index levels were negatively associated with clinical pregnancy and live birth rates in women undergoing IVF with a GnRH antagonist protocol, suggesting that systemic inflammatory burden may impair reproductive success [7]. Likewise, Sucu et al. explored several immunological indexes, including SII, SIRI, and NLR, and found that systemic inflammation influenced oocyte and embryo development in women with unexplained infertility [8]. These recent findings provide further evidence supporting the role of inflammation-related hematological markers in reproductive outcomes and highlight the relevance of our investigation into IG percentage as a novel predictive indicator in IVF.

These results are encouraging for the use of these markers in IVF cases where the immunological environment plays a critical role. Despite the recognized importance of inflammation in reproductive success, the specific contribution of IGs in predicting IVF outcomes remains a gap in the current literature. This paper aims to bridge this gap by reviewing existing studies on hematological inflammatory markers in IVF, with a particular focus on IGs. By doing so, it seeks to shed light on the potential of these markers as predictive tools for IVF success, thereby contributing to the refinement of IVF protocols and enhancing the understanding of the immunological aspects of assisted reproduction.

## 2. Materials and Methods

### 2.1. Study Design and Participants

We conducted this study by retrospectively examining the IVF cycles of 311 distinct patients in the IVF unit of Konya City Hospital between 2017 and 2022. Ethics committee approval was received on 10 August 2022, record number 22/423, from the Health Sciences University Hamidiye Scientific Research Ethics Committee. Written and verbal consent were obtained from everyone who participated in the study. Patients who underwent frozen–thawed embryo transfer; those with comorbid conditions, autoimmune diseases, endometrioma or endometriosis, polycystic ovary syndrome (PCOS); a body mass index (BMI) > 30 kg/m^2^; a history of continuous medication use; or symptoms of acute infection were excluded from the study. Internal medicine consultation was requested from all patients and patients with systemic diseases that would prevent pregnancy were excluded. Data about the patients were accessed from file records. Patient age, paternal age, infertility and marriage duration, and BMI values were obtained from the files and analyzed as demographic data. The basal hormone values, follicle-stimulating hormone (FSH), luteinizing hormone (LH), estradiol (E2), prolactin, progesterone, thyroid-stimulating hormone (TSH), and CBC parameters measured on the 2nd day of menstruation were accessed from computer records. All demographic data, basal hormone values, and inflammatory markers of CBC were compared between groups with and without clinical pregnancy and live birth. Peripheral blood samples were routinely collected on the second day of menstruation while preparing for anesthesia preoperative procedures, before starting gonadotropin treatment. Patients whose routine preoperative examinations were completed were directed to the anesthesia department before the oocyte pick-up (OPU) procedure. Results regarding IVF cycle parameters and pregnancy outcomes were obtained from infertility files. The initial gonadotropin dose, cycle duration, E2 value on hCG day, endometrial thickness on hCG day, total number of oocytes, number of MII oocytes, number of embryos generated, and fertilization rates were compared between the groups with and without clinical pregnancy and live birth. Pregnancies with intrauterine fetal heartbeat were considered as clinical pregnancies, and those who gave birth at 24 weeks or more were considered as live births.

Peripheral blood samples were obtained on the 2nd day of menstruation, prior to gonadotropin stimulation, during the preoperative anesthesia consultation—ensuring that all samples were collected before oocyte retrieval or embryo transfer.

### 2.2. Ovarian Stimulation Protocol

A short flexible antagonist protocol was applied to all patients. On the 2nd or 3rd day of menstruation, after checking the basal hormone profile and ruling out persistent cysts with TVUSG, ovarian stimulation was performed with appropriate doses of 150–225 IU recFSH (Gonal-F^®^, Merck Serono, Rome, Italy) or 150–300 IU hMG (human menopausal gonadotropin) (Menogon^®^, Ferring, GmbH, Kiel, Germany). All patients were stimulated using a standardized short flexible GnRH antagonist protocol; gonadotropin doses were individualized according to ovarian reserve and follicular response to ensure comparable stimulation conditions across participants. When the follicle size reached 13–14 mm, an antagonist (Cetrorelix^®^, Merck Serono, Darmstadt, Germany) was applied flexibly. Folliculometry was continued every 2–3 days with TV USG. Final maturation of oocytes was achieved by applying rechCG (Ovitrelle^®^, Merck-Serono, Rome, Italy) to follicle sizes reaching 17–18 mm. OPU procedure was performed under anesthesia 36 h after hCG application. On the OPU day, sperms were obtained from the partners simultaneously by masturbation. The ICSI procedure was performed on all patients. The best-grade embryos were selected, and fresh embryo transfer was performed on the 3rd day. All patients were administered 600 mg progesterone capsules (Progestan^®^ 200 mg, Koçak Farma, Istanbul, Turkey) intravaginally as luteal support. Beta hCG control was recommended 12 days after embryo transfer.

### 2.3. CBC Laboratory Analysis and Calculations

#### 2.3.1. Sample Collection

Peripheral blood samples of the patients were taken routinely during anesthesia department consultations on the gonadotropin starting day. Blood samples were collected to gel barrier-containing clot-activator tubes (Becton Dickinson and Company (BD) Vacutainer^®^ SST II Advance tube, 5 mL, 13 × 100 mm, Franklin Lakes, NJ, USA), which were used for hormone profile analysis. Additionally, tubes with K2EDTA (BD Vacutainer^®^, 2 mL, 13 × 75 mm, Franklin Lakes, NJ, USA) were used for CBC analysis. The SST II tubes were centrifuged at 1500× *g* for 10 min to obtain serum materials for the hormone profile.

#### 2.3.2. CBC Measurement

We analyzed the hormone profiles of E2, FSH, LH, prolactin, progesterone, and thyroid-stimulating hormones by electrochemiluminescene immunassay on a Cobas 8000 e701 (Roche Diagnostics International Ltd., Basel, Switzerland) apparatus. CBC analyses were performed on the Sysmex XN-1000 Automated Hematology System (Hematology System, Brand, Kobe, Japan). WBC, neutrophil count, lymphocyte count, monocyte count, platelet count, mean platelet volume (MPV), and platelet distribution width (PDW) values were obtained from CBC analyses. In our hospital, the serum IG percentage level is regularly determined as part of a complete blood count via an automated cell analyzer. IGs are sorted in the WBC differential channel of the analyzer. Erythrocytes in the sample are lysed, and leukocytes are fluorescently stained for automated IG detection via flow cytometry, enabling the accurate differentiation of IGs. Software is used to judge WBC type, and IGs are shown in the scattergram. After IG values are obtained, the percentages of IGs are calculated (IG value/WBC value) × 100 automatically by the analyzer.

#### 2.3.3. Calculation Formulas

The following formulas were used for the values obtained through calculations, respectively:

Neutrophil-to-lymphocyte ratio (NLR) = neutrophil count/lymphocyte count;

Platelet-to-lymphocyte ratio (PLR) = platelet count/lymphocyte count;

Monocyte-to-lymphocyte ratio (MLR) = monocyte count/lymphocyte count

Systemic immune–inflammation index (SII) = platelet count × neutrophil count/lymphocyte count.

### 2.4. Statistical Analysis

Data analysis was performed using the IBM SPSS Statistics 26 (IBM Corp., Armonk, NY, USA). Kolmogorov–Smirnov and Shapiro–Wilk tests and histograms were used to assess the conformity of the data to normal distribution. For pairwise comparisons of data conforming to the normal distribution, the independent samples *t*-test was used and results were presented as mean ± standard deviation. For data not conforming to normal distribution, the Mann–Whitney U test was used for pairwise comparisons and results were expressed as median (minimum–maximum). The chi-squared test or the Fisher exact test was used to compare categorical data. Results were expressed as percentages (*n* (%)). Relationships between parameters were analyzed using the Spearman correlation analysis. Receiver operating characteristic (ROC) analysis was performed to evaluate the effectiveness of the systemic inflammatory index in predicting pregnancy and achieving live birth rates. The results of the analysis were reported as sensitivity, specificity, positive predictive value (PPV), negative predictive value (NPV), and area under the curve (AUC). Factors influencing the occurrence of pregnancy were analyzed using logistic regression analysis, and the results were presented with a two-tailed *p*-value at a significance level of 0.05.

## 3. Results

The study was conducted retrospectively with the IVF cycles of a total of 311 patients. Clinical pregnancy occurred in *n* = 103 (33.1%) of 311 patients, and live birth occurred in *n* = 80 (25.7%) of 311 patients. When the demographic data of the study participants were examined, the average female age was 30.67 ± 5.15 years and the average infertility duration was 5.50 ± 3.82 years. Table 1 includes all the mean values of the participants’ demographic data, basal hormone values, inflammatory markers of CBC, and IVF cycle parameters.

When we look at the cycle parameters of the groups with and without clinical pregnancy, endometrial thickness on the hCG day is statistically significantly higher in the clinical pregnancy group (10.39 ± 1.44 mm vs. 9.96 ± 1.70 mm, *p* = 0.026). The number of MII oocytes was higher in the clinical pregnancy group (*n* = 6.00 vs. *n* = 5.00, *p* = 0.014). The number of embryos generated was statistically significantly higher in the clinical pregnancy group (*n* = 4.00 vs. *n* = 3.00, *p* = 0.015). When we examined the inflammatory parameters in the CBC of the groups with and without clinical pregnancy, WBC and SII values were found to be higher in the group with clinical pregnancy (*p* = 0.004 and *p* = 0.001, respectively). The IG percentage was found to be lower in the clinical pregnancy group (*p* = 0.001). The IG percentage was also statistically significantly lower in the live birth group (0.02 ± 0.01 vs. 0.04 ± 0.01, *p* = 0.001). Table 2 and Table 3 show the demographic data, IVF cycle results, and inflammatory parameters on CBC in groups with and without clinical pregnancy and live births.

Table 4 shows the correlations of IVF outcome variables, including embryo grade, live birth rates, and CBC parameters. A strong negative correlation was found between IG percentage and live birth rates (r = −0.554, *p* = 0.001), indicating that lower IG% values were associated with higher clinical pregnancy and live birth rates. A similar strong negative correlation was observed between IG percentage and clinical pregnancy rates (r = −0.523, *p* = 0.001). Correlations with other variables were generally weak and some were not statistically significant.

Logistic regression analysis was performed to determine the factors influencing clinical pregnancy positivity. The goodness-of-fit measures of the model show a deviance of 267 and an AIC of 287. McFadden’s R-squared value was 0.323 and Nagelkerke’s R-squared value was 0.468. According to the model, embryo quality has a significant effect on the occurrence of clinical pregnancy. In particular, the difference between Grade 1 and Grade 3 embryo quality significantly increased the chance of clinical pregnancy with an odds ratio (OR) value of 6.55 (95% CI: 1.973–21.76, *p* = 0.002). Similarly, the difference between grade 2 and grade 3 embryo quality was also significant with an OR value of 3.39 (95% CI: 1.023–11.26, *p* = 0.046). IG percentage (OR = 0.40, 95% CI: 0.317–0.51, *p* < 0.001) significantly affected clinical pregnancy outcomes. Each millimeter increase in endometrial thickness measured on the hCG day slightly increased the chance of success (OR: 1.20, 95% CI: 1.004–1.44, *p* = 0.044). On the other hand, some determinants, such as the number of SII and MII oocytes, did not have a statistically significant effect on clinical pregnancy outcome (Table 5). Although the model demonstrated significant predictive performance (deviance = 267, AIC = 287), its explanatory power was moderate (McFadden’s R^2^ = 0.323, Nagelkerke’s R^2^ = 0.468), suggesting that while IG% and embryo quality are important predictors, other biological and clinical variables likely influence IVF outcomes beyond the scope of this model.

**Figure 1:**
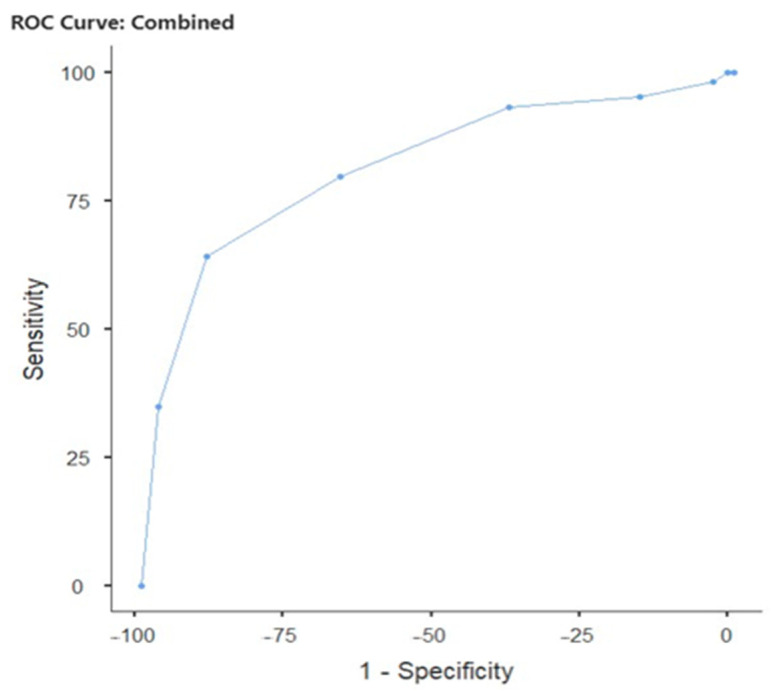
Diagnostic performance and optimal cut-off points for pregnancy prediction based on IG percentage.

**Figure 2:**
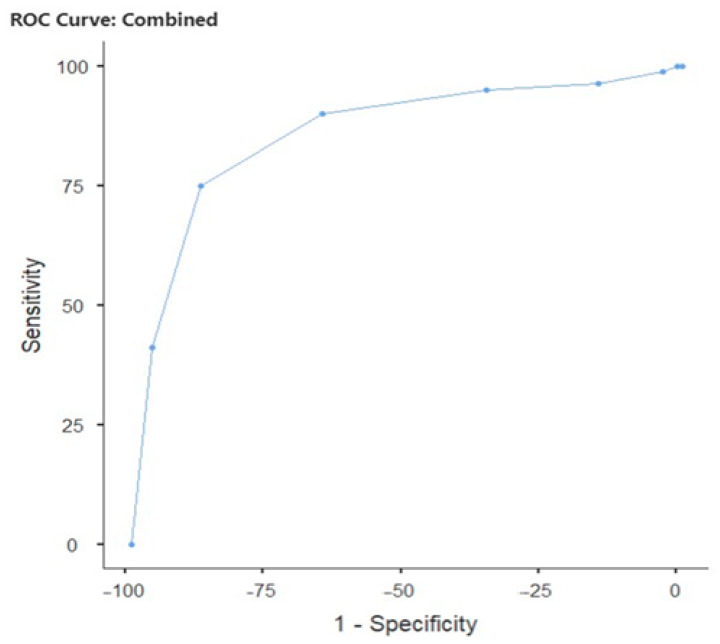
Live birth prediction performance of IG percentage in IVF patients. Cut-Off = 0.02; AUC: 0.860; 95% CI: 0.81–0.90; sensitivity = 75%; and specificity = 87.45%.

Table 6 shows the diagnostic performance of IG percentage in predicting clinical pregnancy with two different cut-off points: at the cut-off point of 0.02, sensitivity was 64.08%, specificity was 88.94%, PPV (positive predictive value) was 74.16%, NPV (negative predictive value) was 83.33%, Youden’s index was 0.530, and AUC (Area under the curve) was 0.815 (Figure 1). Table 7 shows the capacity of IG percentage to predict live births with a cut-off point of 0.02. Accordingly, sensitivity was 75.00%, specificity was 87.45%, PPV was 67.42%, NPV was 90.99%, Youden’s index was 0.624, and AUC was 0.860 (Figure 2).

## 4. Discussion

In this study, we explored the clinical pregnancy and live birth rates in 311 IVF cycles of 311 distinct patients using the short antagonist protocol, examining the relationship between pregnancy outcomes and various inflammatory markers obtained from CBC tests. Our findings reveal a statistically significantly lower percentage of IG in the live birth group compared to the non-live-birth group within the IVF context, which aligns with the notion that a lower inflammatory state might favor reproductive success. These findings revealed that, except for the percentage of IG, none of the inflammatory markers showed a significant correlation with clinical pregnancy and live birth rates. This study is a pioneering one in investigating the impact of IG percentage in the IVF field and may provide new insights into how inflammation can affect IVF outcomes, emphasizing its significant impact on reproductive success. However, the explanatory power of our regression model (McFadden’s R^2^ = 0.323, Nagelkerke’s R^2^ = 0.468) was moderate, indicating that although IG% and embryo quality significantly contribute to IVF success, multifactorial biological mechanisms also play essential roles that extend beyond the parameters included in the current model.

The overproduction of chemokines and cytokines hinders neutrophil movement to the inflammation site, leading to the release of immature neutrophils into the bloodstream as substitutes for the shortfall of functioning neutrophils [9]. However, accurately measuring IGs remains difficult. Our study concentrated on the automated counting of these immature cells [10]. Elevated IG% levels may thus indicate systemic inflammatory activation driven by cytokine-mediated bone marrow stimulation. Increased IG release can reflect an overactive immune state, which might interfere with endometrial receptivity through the dysregulation of local cytokine networks, impaired decidualization, or altered vascularization. Such an imbalance may hinder trophoblast invasion and embryo implantation, consistent with our finding that higher IG% values were associated with lower clinical pregnancy and live birth rates.

Inflammation plays a significant role in human pregnancy, particularly in the success of implantation and the progression of pregnancy [11,12]. During pregnancy, the maternal immune system undergoes a unique adaptation process to tolerate the semi-allogenic embryo. Regulatory T cells (Tregs) and uterine natural killer (uNK) cells contribute to maintaining this balance by modulating cytokine responses and supporting trophoblast invasion [13,14,15]. In our study, we observed that a lower percentage of IGs was strongly associated with improved clinical pregnancy and live birth outcomes following IVF. This finding suggests that a reduced systemic inflammatory state, as reflected by lower levels of circulating IGs, may create a more favorable environment for embryo implantation and development.

Although the percentage of IGs has not been studied in the field of IVF, there are many studies conducted in various areas of obstetrics and gynecology. Dogru et al. evaluated the predictive value of the delta neutrophil index (DNI) and other blood parameters on perinatal outcomes in pregnant women with systemic lupus erythematosus [16]. The results showed that NLR, PLR, and DNI were significantly higher in SLE patients compared to the healthy controls. DNI had predictive values for small for gestational age (SGA) and stillbirth, with ROC and AUC values of 0.666 and 0.731, respectively. SLE patients had higher rates of cesarean section, preeclampsia, earlier delivery, lower birth weight, higher SGA, and stillbirth rates. A study investigated the predictive value of the DNI for histological chorioamnionitis. Findings showed that maternal DNI and SII were significantly higher in histological chorioamnionitis cases, predicting neonatal intensive care unit admissions [17]. In contrast to these studies, Dal et al. investigated the role of the serum DNI and other inflammatory markers in hyperemesis gravidarum (HEG) [18]. The results showed no significant difference in the DNI, platelet-to-lymphocyte ratio, monocyte-to-lymphocyte ratio, and systemic inflammation index between the HEG patients and healthy pregnant women. However, the neutrophil count and NLR were higher in the HEG group. The study concluded that DNI does not reflect the presence and severity of HEG.

Recent studies have similarly emphasized the connection between systemic inflammation and IVF outcomes. Li et al. reported that elevated systemic immune–inflammation index (SII) values were negatively associated with clinical pregnancy and live birth rates in women undergoing IVF [7]. Sucu et al. also demonstrated that hematological inflammation indices such as SII, SIRI, and NLR influenced oocyte maturation and embryo development in unexplained infertility cases [8]. These findings support our results and highlight that systemic inflammatory activation may impair reproductive performance through both the ovarian and endometrial pathways.

It appears that while various inflammatory markers are integral to understanding the underlying mechanisms of IVF outcomes, their predictive value varies. For instance, the study by Ozgu-Erdinc et al. focused on the predictive role of inflammatory hematological markers in IVF success, highlighting the significance of PLR in particular [1]. The positive hCG group had a statistically lower PLR when compared to the hCG (-) group (*p* = 0.02). In the ROC analysis, PLR was significant in predicting positive hCG (*p* = 0.028). However, when they added other factors to the model, only age and MII oocyte count were successful in predicting pregnancy outcomes in a logistic regression analysis, and not the inflammatory markers of CBC. Another study emphasized the relationship between CBC inflammation markers and IVF outcomes in non-obese women with unexplained infertility (UI), showing that there was a positive association between lymphocyte count and fertilization rate and a negative association between PLR and implantation among UI patients. None of the inflammatory markers of CBC were predictive for clinical pregnancy, take-home baby, and clinical and biochemical abortion rates among non-obese UI patients [2]. Xin Li et al. illustrated that the highest quartiles of SII were negatively associated with the biochemical pregnancy rate, clinical pregnancy rate, and live birth rate. Conversely, a positive association was found with the early pregnancy loss rate, albeit with no statistically significant difference (*p* > 0.05) [19].

There were some limitations in our study; firstly, it was retrospective in nature, with a small sample size. This limitation could affect the comprehensiveness of the analysis regarding factors influencing IVF success. Additionally, since our study is a single-doctor, single-center study, it does not provide information about the general population. It should also be noted that inflammatory hematological markers such as IG%, NLR, and SII are dynamic and can fluctuate in response to transient physiological or environmental factors including minor infections, psychological stress, or hormonal variations. Such fluctuations may introduce variability that cannot be fully controlled in a retrospective design. Therefore, further studies are needed to validate the role of the IG percentage in predicting clinical and live birth outcomes as indicators of IVF success.

In conclusion, we found in our study that a low IG percentage was associated with clinical pregnancy and live birth rates. This suggests that IG% can serve as a simple, rapid, and cost-effective biomarker to evaluate systemic immune status prior to IVF treatment. Nevertheless, as inflammation plays a critical role in implantation and pregnancy maintenance, IG% should be considered within a multifactorial predictive framework that incorporates both clinical and immunological factors. Further research, particularly prospective studies, is required to validate these findings and potentially integrate them into clinical practice, developing a predictive framework for IVF success.

## Figures and Tables

**Table 1:** Demographic, clinical, and laboratory characteristics of the women who underwent in vitro fertilization.

	**Mean ± SD**	**Median (Min–Max)**
Age (years)	30.67 ± 5.15	30.00 (18.00–44.00)
Partner’s age (years)	33.64 ± 5.84	33.00 (20.00–58.00)
Marriage duration (years)	5.88 ± 4.26	5.00 (0.00–21.00)
Infertility duration (years)	5.50 ± 3.82	4.00 (0.30–21.00)
Gravida (*n*)	0.15 ± 0.55	0.00 (0.00–5.00)
Parity (*n*)	0.08 ± 0.30	0.00 (0.00–2.00)
Number of living children (*n*)	0.08 ± 0.30	0.00 (0.00–2.00)
BMI (kg/m^2^)	26.05 ± 4.61	25.59 (15.38–45.49)
E2 (ng/L)	50.20 ± 35.10	44.08 (1.80–316.50)
FSH (U/L)	7.65 ± 3.18	7.27 (0.20–26.87)
LH (U/L)	4.74 ± 2.66	4.35 (0.06–27.42)
TSH (mU/L)	2.38 ± 1.15	2.14 (0.00–6.91)
Prolactin (microgram/L)	13.91 ± 8.61	11.70 (0.29–69.51)
Progesterone (microgram/L)	0.45 ± 0.40	0.40 (0.00–3.97)
WBC	8.43 ± 2.25	8.19 (3.15–17.86)
MPV	10.37 ± 1.29	10.30 (7.20–21.60)
PDW	13.68 ± 4.51	13.10 (8.80–49.90)
IG (%)	0.04 ± 0.02	0.04 (0.01–0.08)
NLR	2.63 ± 1.68	2.18 (0.34–15.80)
PLR	131.37 ± 63.42	120.30 (35.58–640.43)
MLR	0.28 ± 0.17	0.25 (0.05–1.53)
SII	720.19 ± 477.94	626.52 (104.69–3893.79)
Gonadotropin starting dose (IU)	196.34 ± 55.63	187.00 (75.00–375.00)
Induction duration (days)	8.74 ± 1.52	9.00 (4.00–15.00)
E2 value on hCG day (ng/L)	2463.85 ± 1474.45	2172.00 (250.00–17,750.00)
Endometrial thickness on hCG day (mm)	10.11 ± 1.63	10.00 (6.00–18.00)
Total number of oocytes	9.07 ± 5.91	8.00 (1.00–40.00)
Number of metaphase II oocytes	6.73 ± 4.80	6.00 (1.00–36.00)
Number of embryos	4.26 ± 3.33	4.00 (1.00–23.00)
Fertilization rate	67.79 ± 25.47	67.00 (9.09–100.00)

BMI: Body mass index; E2: estradiol; FSH: follicle-stimulating hormone; LH: luteinizing hormone; TSH: thyroid-stimulating hormone; WBC: white blood cell; MPV: main platelet volume; PDW: platelet distribution width; IG: immature granulocyte; NLR: neutrophil-to-lymphocyte ratio; PLR: platelet-to-lymphocyte ratio; MLR: monocyte-to-lymphocyte ratio; SII: systemic inflammatory index.

**Table 2:** Comparison of demographic, clinical, and laboratory parameters in IVF patients according to clinical pregnancy results.

		Pregnancy-Positive (*n* = 103)	Pregnancy-Negative (*n* = 208)	*p*-Value
Demographic characteristics (*n* = 311)				
Age (years)		30.25 ± 4.56	30.88 ± 5.43	0.313
Partner’s age (years)		33.14 ± 4.72	33.89 ± 6.32	0.282
Marriage duration (years)		4 (0.00–17.00)	5.00 (0.00–21.00)	0.098
Infertility duration (years)		4 (0.50–17.00)	5 (0.30–21.00)	0.071
Gravida (*n*)		0 (0.00–4.00)	0 (0.00–5.00)	0.507
Parity (*n*)		0 (0.00–2.00)	0 (0.00–2.00)	0.744
Number of living children		0 (0.00–2.00)	0 (0.00–2.00)	0.744
BMI (kg/m^2^)		26.01 ± 4.47	26.08 ± 4.69	0.890
Infertility type (*n*/%)	Unexplained	31 (30.1%)	67 (32.2%)	0.755
	Poor ovarian reserve	29 (28.2%)	69 (33.2%)	
	Male factor	40 (38.8%)	66 (31.7%)	
	Tubal factor	1 (1.0%)	3 (1.4%)	
	Male factor + poor ovary reserve	2 (1.9%)	3 (1.4%)	
Laboratory values (*n* = 311)				
E2 (ng/L)		50.52 ± 32.57	50.05 ± 36.37	0.912
FSH (U/L)		7.50 ± 2.92	7.72 ± 3.31	0.565
LH (U/L)		4.85 ± 2.07	4.69 ± 2.91	0.612
TSH (mU/L)		2.44 ± 1.18	2.35 ± 1.13	0.511
Prolactin (microgram/L)		14.26 ± 8.66	13.74 ± 8.60	0.617
Progesterone (microgram/L)		0.38 (0.00–1.05)	0.40 (0.00–3.97)	0.297
WBC		8.95 ± 2.47	8.18 ± 2.10	0.004
MPV		10.33 ± 0.98	10.39 ± 1.42	0.685
PDW		12.70 (8.80–19.50)	13.20 (9.40–4.90)	0.055
IG (%)		0.02 ± 0.01	0.04 ± 0.01	0.001
NLR		3.02 ± 1.70	2.44 ± 1.64	0.004
PLR		135.79 ± 46.92	129.19 ± 70.17	0.389
MLR		0.30 ± 0.10	0.27 ± 0.18	0.147
SII		711.04 (243.14–3595.13)	581.62 (104.69–3893.78)	0.001
IVF-related parameters (*n* = 311)				
Gonadotropin starting dose (IU)		191.53 ± 55.56	198.71 ± 55.64	0.285
Induction duration (days)		8.6311 ± 1.21	8.7885 ± 1.64	0.390
E2 value on hCG day (ng/L)		2343.03 ± 1121.59	2523.67 ± 1620.18	0.310
Endometrial thickness on hCG day (mm)		10.39 ± 1.44	9.96 ± 1.70	0.026
Total number of oocytes		8.00 (1.00–27.00)	8.00 (1.00–40.00)	0.090
Number of metaphase II oocytes		6.00 (1.00–24.00)	5.00 (1.00–36.00)	0.014
Number of embryos		4.00 (1.00–21.00)	3.00 (1.00–23.00)	0.015
Fertilization rate		66.67 (10.00–100.00)	67.00(9.09–100.00)	0.657
Embryo grade	Grade 1	58 (56.3%)	69 (33.2%)	0.001
	Grade 2	41 (39.8%)	101 (48.6%)	
	Grade 3	4 (3.9%)	38 (18.3%)	

BMI: Body mass index; E2: estradiol; FSH: follicle-stimulating hormone; LH: luteinizing hormone; TSH: thyroid-stimulating hormone; WBC: white blood cell; MPV: main platelet volume; PDW: platelet distribution width; IG: immature granulocyte; NLR: neutrophil-to-lymphocyte ratio; PLR: platelet-to-lymphocyte ratio; MLR: monocyte-to-lymphocyte ratio; SII: systemic inflammatory index.

**Table 3:** Comparison of demographic, clinical, and laboratory parameters in IVF patients according to live birth results.

			Live Birth-Positive (*n* = 80)	Live Birth-Negative (*n* = 231)	*p*-Value
Demographic characteristics (*n* = 311)					
Age (years)			29.94 ± 4.34	30.93 ± 5.40	0.140
Partner’s age (years)			32.94 ± 5.00	33.89 ± 6.10	0.210
Marriage duration (years)			4.00 (0.00–17.00)	5.00 (0.00–21.00)	0.119
Infertility duration (years)			4.00 (0.00–17.00)	5.00 (0.00–21.00)	0.088
Gravida (number)			0.00 (0.00–4.00)	0.00 (0.00–5.00)	0.343
Parity (number)			0.00 (0.00–2.00)	0.00 (0.00–2.00)	0.775
Number of living children			0.00 (0.00–2.00)	0.00 (0.00–2.00)	0.775
BMI (kg/m^2^)			26.25 ± 4.74	25.95 ± 4.66	0.621
Infertility type (*n*/%)		Unexplained	24 (30.0%)	74 (32.0%)	0.933
		Poor ovarian reserve	24 (30.0%)	74 (32.0%)	
		Male factor	29 (36.3%)	77 (33.3%)	
		Tubal factor	1 (1.3%)	3 (1.3%)	
		Male factor + poor ovary reserve	2 (2.5%)	3 (1.3%)	
Laboratory values (*n* = 311)					
E2 (ng/L)			44.77 (11.80–316.50)	43.73 (1.80–315.53)	0.325
FSH (U/L)			7.52 ± 2.88	7.69 ± 3.28	0.685
LH (U/L)			4.82 ± 2.07	4.71 ± 2.84	0.771
TSH (mU/L)			2.45 ± 1.21	2.35 ± 1.13	0.516
Prolactin (microgram/L)			14.69 ± 9.09	13.64 ± 8.44	0.346
Progesterone (microgram/L)			0.36 (0.00–1.05)	0.41 (0.00–3.97)	0.431
WBC			9.03 ± 2.48	8.23 ± 2.13	0.006
MPV			10.36 ± 1.02	10.38 ± 1.37	0.896
PDW			13.13 ± 2.20	13.88 ± 5.06	0.199
IG (%)			0.02 ± 0.01	0.04 ± 0.01	0.001
NLR			2.93 ± 1.66	2.53 ± 1.67	0.062
PLR			122,23 (50.45–321.57)	117.78 (35.58–640.43)	0.200
MLR			0.26 (0.10–1.01)	0.24 (0.05–1.53)	0.239
SII			709.04 (243.14–3595.14)	596.57 (104.69–3893.79)	0.007
IVF-related parameters (*n* = 311)					
Gonadotropin starting dose (IU)			193.81 ± 52.98	197.21 ± 56.61	0.638
Induction duration (days)			8.60 ± 1.15	8.78 ± 1.62	0.352
E2 value on hCG day (ng/L)			2392.78 ± 1141.06	2488.46 ± 1574.99	0.618
Endometrial thickness on hCG day (mm)			10.44 ± 1.46	9.99 ± 1.67	0.034
Total number of oocytes			8.00 (1.00–27.00)	8.00 (1.00–40.00)	0.339
Number of metaphase II oocytes			6.00 (1.00–24.00)	5.00 (1.00–36.00)	0.053
Number of embryos			4.00 (1.00–21.00)	3.00 (1.00–23.00)	0.023
Fertilization rate (%)			71.43 (10.00–100.00)	67.00 (9.09–100.00)	0.849
Embryo grade (*n*)	Grade 1		44 (55.0%)	83 (35.9%)	0.001
	Grade 2		34 (42.5%)	108 (46.8%)	
	Grade 3		2 (2.5%)	40 (17.3%)	

BMI: Body mass index; E2: estradiol; FSH: follicle-stimulating hormone; LH: luteinizing hormone; TSH: thyroid-stimulating hormone; WBC: white blood cell; MPV: main platelet volume; PDW: platelet distribution width; IG: immature granulocyte; NLR: neutrophil-to-lymphocyte ratio; PLR: platelet-to-lymphocyte ratio; MLR: monocyte-to-lymphocyte ratio; SII: systemic inflammatory index.

**Table 4:** Correlations of IVF outcome variables including embryo grade, live birth rates, and laboratory findings.

	IG (%)	Embryo Grade	Live Birth	Pregnancy	Total Oocytes	Number of Embryos	Fertilization Rate
IG (%)	1						
Embryo Grade	0.148 *p* = 0.009	1					
Live Birth	0.554 *p* = 0.001	0.209 *p* = 0.001	1				
Clinical Pregnancy	0.523 *p* = 0.001	0.255 *p* = 0.001	0.836 *p* = 0.001	1			
Total Oocytes	−0.092 *p* = 0.105	−0.182 *p* = 0.001	−0.054 *p* = 0.340	−0.096 *p* = 0.090	1		
Embryo Cleavage Rate	−0.084 *p* = 0.137	−0.307 *p* < 0.001	−0.130 *p* = 0.022	−0.138 *p* = 0.015	0.705 *p* = 0.001	1	
Fertilization Rate	0.069 *p* = 0.226	−0.040 *p* = 0.481	−0.011 *p* = 0.849	0.025 *p* = 0.657	−0.291 *p* = 0.001	0.250 *p* = 0.001	1

**Table 5:** Logistic regression analysis of in vitro fertilization success determinants for clinical pregnancy status.

Predictor	Estimate	SE	*p*	Odds Ratio	Lower	Upper
Intercept	−2.19	1.24	0.078	0.11	0.009	1.27
Embryo grade: Grade 1–Grade 3	1.88	0.61	0.002	6.55	1.973	21.76
Embryo grade: Grade 2–Grade 3	1.22	0.61	0.046	3.39	1.023	11.26
WBC	−0.24	0.18	0.177	0.78	0.546	1.11
IG (%)	−0.91	0.12	0.001	0.40	0.317	0.51
SII	−0.0001	0.00	0.773	1.00	0.998	1.00
Endometrial thickness on hCG day (mm)	0.18	0.09	0.044	1.20	1.004	1.44
Number of metaphase II oocytes	0.04	0.05	0.473	1.04	0.932	1.16
Number of embryos	−0.05	0.08	0.521	0.94	0.810	1.11

**Table 6:** Diagnostic performance metrics of IG percentage for predicting clinical pregnancy.

Cut-Off Point	Sensitivity (%)	Specificity (%)	PPV (%)	NPV (%)	Youden’s Index	AUC
0.02	64.08%	88.94%	74.16%	83.33%	0.530	0.815

**Table 7:** Diagnostic performance metrics of IG percentage for predicting live births.

Cut-Off Point	Sensitivity (%)	Specificity (%)	PPV (%)	NPV (%)	Youden’s Index	AUC
0.02	75.00	87.45	67.42	90.99	0.624	0.860

## Data Availability

Data is available upon request from the corresponding author.
